# Robotic versus open ventral hernia repair (ROVHR): a randomized controlled trial protocol

**DOI:** 10.1007/s10029-025-03299-7

**Published:** 2025-03-04

**Authors:** Alvaro C. Carvalho, Kimberly P. Woo, Ryan C. Ellis, Chao Tu, Benjamin T. Miller, Ajita S. Prabhu, Michael J. Rosen, David M. Krpata, Clayton C. Petro, Lucas R. Beffa

**Affiliations:** 1 Department of Surgery, Cleveland Clinic Center for Abdominal Core Health, Cleveland Clinic Foundation, 9500 Euclid Ave, Cleveland, OH USA; 2 Quantitative Health Sciences, Cleveland Clinic, Cleveland, OH USA

**Keywords:** Ventral hernia, Retromuscular repair, Robotic ventral hernia, Open ventral hernia

## Abstract

**Background:**

Robotic retromuscular hernia repair has proven to be feasible and safe but lacks randomized data to demonstrate significant clinical benefit. The majority of current comparative studies published have been case series, retrospective studies, systematic reviews, or large registry data, all of which have significant limitations and bias (Bittner et al. in Surg Endosc 32:727–734. 10.1007/s00464-017-5729-0, 2018; Bracale et al in Hernia 25:1471–1480. 10.1007/s10029-021-02487-5, 2021; Carbonell in Ann Surg 267:210–217. 10.1097/SLA.0000000000002244, 2018; (Warren et al. in Surg Endosc. 10.1007/s00464-024-11202-1, 2024; Dewulf et al in BJS Open 6:zrac057. 10.1093/bjsopen/zrac057, 2022; Maskal and Beffa in Surg Clin N Am 103:977–991. 10.1016/j.suc.2023.04.007, 2023). It was only recently that the first randomized trial was conducted by Warren et al. comparing open and robotic retromuscular hernia repairs with synthetic mesh (Warren et al. in Surg Endosc. 10.1007/s00464-024-11202-1, 2024). The data currently available has yielded inconsistent outcomes leaving significant knowledge gaps for clinical decision making. Reduced length of stay for robotic retromuscular repairs has been a consistently proven outcome, however, and therefore, we hypothesized that robotic retromuscular hernia repairs would be superior to open retromuscular hernia repair by reducing length of stay in the hospital by 24 h (Carbonell in Ann Surg 267:210–217. 10.1097/SLA.0000000000002244, 2018).

**Methods:**

The Institutional Review Board at all participating sites has approved this protocol. This trial has been registered on clinicaltrials.gov (NCT: 05472987). The ROVHR trial is a registry-based, multicenter, double-blinded randomized trial. The primary hypothesis is robotic retromuscular hernia repairs is superior to open retromuscular hernia repairs by reducing length of stay by at least 24 h. Secondary outcomes include 30-day wound morbidity, readmissions, opioids prescribed and consumed, NRS-11 pain scores obtained daily for the 5 first days after surgery, PROMIS-3a Pain Intensity survey, and patient reported outcomes including Hernia-Related Quality of Life (HerQLes), and EuraHS. Additionally, direct operating room costs will be compared.

**Discussion:**

Based existing literature, we designed a randomized trial with a primary endpoint to determine if robotic retromuscular hernia repairs reduce length of in hospital stay by at least 24 h compared to open retromuscular hernia repairs. This study will add high-level of evidence providing evidence-based outcomes for clinical decision making.

**Trial registration:**

NCT05472987. Registered on July 20, 2022.

## Introduction

### Background and rationale

Open surgery to correct fascial defects in the abdominal wall has long been considered the gold standard for abdominal wall reconstruction and complex hernia repair, including component separation techniques [7]. Retromuscular hernia repairs for these complex hernias has been favored and endorsed by societal [8, 9]. As surgical innovation and technology advanced, the robotic platform enable surgeons to perform the same a minimally invasive approach to abdominal wall reconstruction. Essentially converting a surgery that traditionally required an open incision to a minimally invasive one [6].

The selection of hernia repair technique is complex and individualized. The techniques available to the hernia surgeons are vast and include open, endoscopic, laparoscopic, hybrid, and robotic techniques, each with their own set of risks and benefits. The data driving surgeon decision based on approach is low quality leaving the surgeon to choose the type of repair based on the hernia morphology, patient co-morbidities, hospital resources, and surgeon training. The need to compare these various surgical techniques remains an ongoing investigative effort with aims to identify where the benefit lies for each individual technique to the patient. With the growth of robotic hernia surgery in the United States, these techniques require evaluation [6, 10].

Therefore, we designed a randomized trial based on previously published retrospective and prospective data that consistently favor robotic retromuscular repair when compared to open retromuscular repairs by reducing in-hospital length of stay.

### Explanation for the choice of comparators

The control arm will be open ventral retromuscular hernia repairs with synthetic mesh. The intervention arm will be robotic retromuscular ventral hernia repair with synthetic mesh.

## Objectives

**Primary Outcome:** To determine if robotic retromuscular ventral hernia repair can reduce in hospital length of stay (LOS) by at least 24 h compared to open retromuscular ventral hernia repair.

**Secondary Outcomes:** Baseline, 30-day, and one-year clinical outcomes include: surgical site occurrence (SSO); surgical site infection (SSI) (superficial or deep); surgical site occurrence requiring procedure (SSOPI); reoperation; mesh excision and other peri-operative complications (ileus, VTE, UTI, etc.). Additionally, readmissions, opioids prescribed and consumed and NRS-11 pain scores obtained daily while standing for the first 5 days after surgery will be recorded. Baseline, 30-day, and one year patient reported outcomes include PROMIS 3a SF, Hernia-related Quality of Life (HerQLes), and EuraHS. Direct costs and operating time will be recorded and compared between the two arms.

After one year, hernia recurrence will be reported via hernia recurrence inventory with or without physical exam or CT-scans following a pragmatic composite recurrence definition.

### Trial design

This is a multi-center, registry-embedded, double-blinded, randomized controlled trial. Study data will be captured in the Abdominal Core Health Quality Collaborative (ACHQC) registry and supplemented with Research Electronic Data Capture (REDCap®) for variables outside of the ACHQC. These include LOS measured in hours, operating time, direct cost, NRS 11 pain scores, and EuraHS scores.

## Methods: Participants, interventions and outcomes

### Study setting

Cleveland Clinic is the primary site, with additional participating sites including University of Tennessee, University of Florida, and Prisma Health.

### Eligibility criteria

*Inclusion criteria*:

- Adults ≥ 18 years old.
- Midline ventral hernia defects ranging from 7 to 15 cm in greatest width as measured by pre-operative CT scan without Valsalva maneuver.
- Non-emergent case
- Body mass index (BMI) less than or equal to 45
- Patient deemed either a robotic or an open candidate by the operating surgeon

*Exclusion criteria*:

- Patients 17 years old or younger
- Prisoners
- Emergent cases
- BMI greater than 45
- Hernia defects less than 7 cm or greater than 15 cm in width as measured by pre-operative CT scan without Valsalva

### Interventions

#### Intervention description

Patients will be enrolled in an elective setting who are planning to undergo ventral hernia repair surgery at Cleveland Clinic main campus or the other participating sites. After appropriate documentation of inclusion and exclusion criteria, informed consent will be obtained.

**Open retromuscular mesh hernia repair (Control arm):** during open surgery the previous midline scar, if present, is excised, however, if not present than a midline laparotomy will be performed. Adhesiolysis will then be performed to free the abdominal wall. Once free, a posterior rectus sheath release will be performed bilaterally. If the surgeon determines rectus sheath alone is inadequate to facilitate hernia repair than a transversus abdominis release (TAR) is performed. This is performed by dividing the transversus abdominis muscle and developing the pre-peritoneal plane to the psoas muscle bilaterally. The neurovascular perforators in the retro-rectus space are identified and preserved. Next, the posterior sheath is closed in the midline with absorbable sutures and a piece of permanent synthetic mesh is placed into the retromuscular pocket. Fixation of the mesh is at the discretion of the surgeon. The anterior rectus sheath is then closed with either a running suture or figure of eight interrupted sutures. Drains are left at the discretion of the surgeon.

**Robotic retromuscular mesh hernia repair (Intervention arm):** In brief, robotic ports are placed intra-abdominally and adhesiolysis completed. If a retro-rectus repair alone is deemed sufficient, then either a single-dock retro-rectus repairs is performed or an enhanced-view totally extra-peritoneal (eTEP) approach can be utilized based on surgeon judgement and expertise. A trans-abdominal single dock retro rectus repair begins with incision of the ipsilateral posterior rectus sheath at least 5 cm lateral to the edge of the fascial defect and developing the ipsilateral retromuscular plane to the linea alba. The preperitoneal space is then entered at the midline by incising the posterior rectus sheath posterior to the linea alba. The peritoneum is kept intact and used to cross over to the opposite side of the abdomen in the midline. The contralateral posterior rectus sheath is then incised, and dissection continued to the semilunar line on the opposite side of the patient. The midline posterior sheath and/or peritoneum is closed with absorbable suture and the anterior fascial defect is closed with a running barbed suture. A permanent synthetic mesh is then placed into the retromuscular pocket and the initial incision in the ipsilateral posterior sheath is closed to completely exclude the mesh from the abdominal cavity. If an eTEP is performed, the dissection is similar to the single dock retrorectus repair, however, the robotic ports are all placed within the retromuscular space, and only bilateral retrorectus dissections are performed without entering into the abdominal cavity. The peritoneum may be used in the midline as tissue coverage if needed.

Alternatively, if a robotic TAR is thought to be needed after assessing the hernia following adhesiolysis, then the surgeon will proceed with incision of the contralateral posterior sheath 1 cm from the linea alba and developing that retro-rectus space to the semilunar line. The transversus abdominis muscle is then divided and dissection carried laterally to the psoas muscle in the pre-peritoneal plane. Three robotic ports are then placed in the retromuscular plane, and a similar dissection is performed on the contralateral abdominal wall. The posterior rectus sheath is then reapproximated in the midline with running absorbable suture. The anterior fascia is then closed with running absorbable suture. A permanent synthetic mesh is then deployed against the closed posterior layer. Drains are left at the discretion of the surgeon. 

### Criteria for discontinuing or modifying allocated interventions

The primary endpoint will be analyzed via intention-to-treat. In the case that a patient is randomized, but the allocated procedure is not completed for technical reasons, they will be analyzed based on randomization. If patients require reoperation with conversion to the alternative treatment arm, all subsequent adverse events will be attributed to the randomized allocation.

### Strategies to improve adherence to interventions

**Fig. 1:**
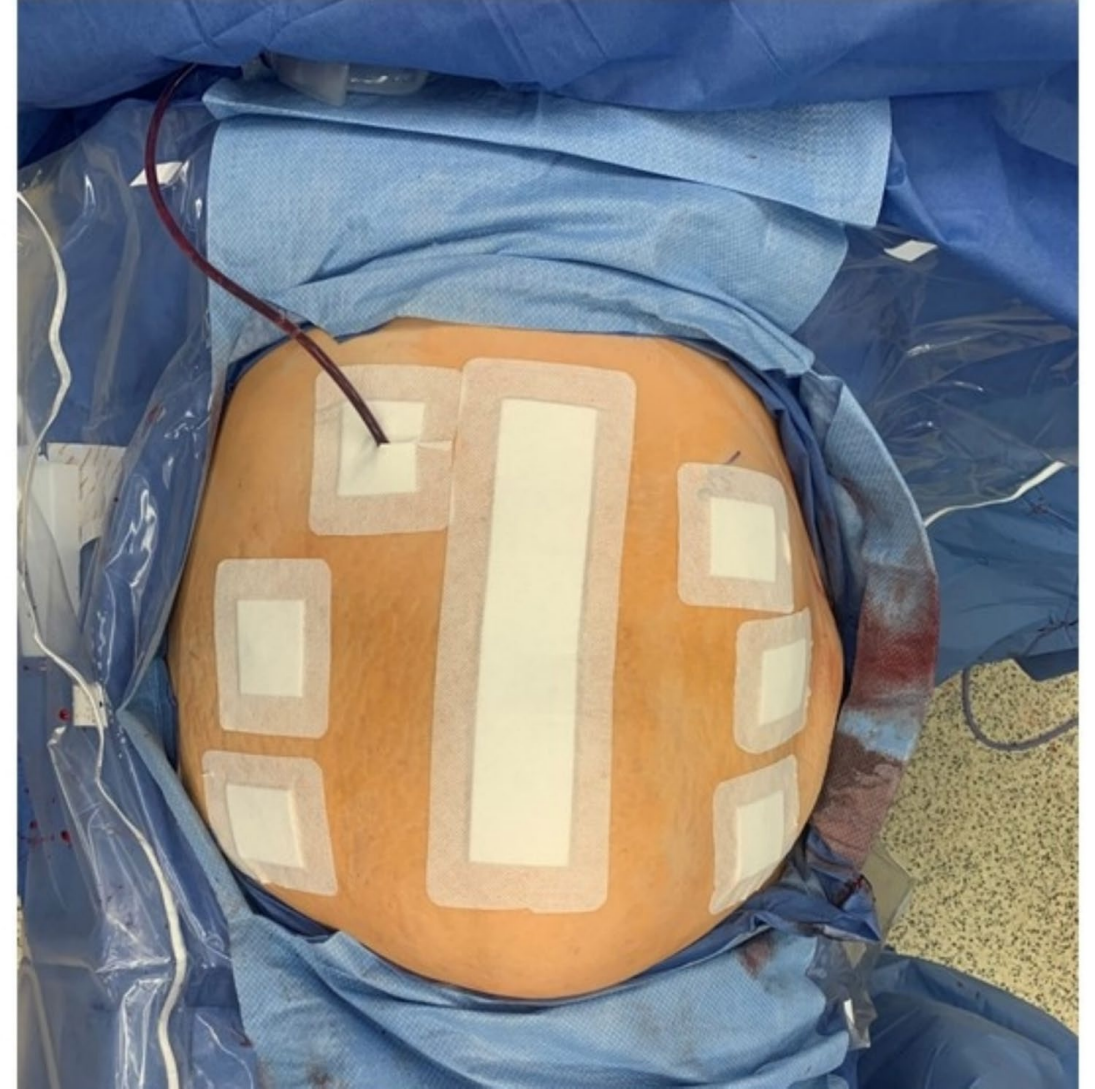
Dressing diagram. Every patient receives 6 island and 1 midline Primapore dressing, regardless of surgery performed

LOS is our primary outcome, and we recognize it can be easily manipulated, therefore we planned to take every possible precaution to limit bias. Post-operatively, patients will remain blinded to the intervention with the use of opaque bandages as if a midline incision and six robotic port sites were made (Fig. 1). As an additional precaution an abdominal binder will be placed on all patients and left in place for the duration of hospital stay. Patients will be discharged by a blinded assessor who is a fellowship trained hernia surgeon, but not an enrolling surgeon in the trial. On postoperative day 1, both the patient and the blinded assessor surgeon will be asked to postulate if the surgery was robotic or open. Discharge criteria was established to standardize the two groups, which consist of the following: tolerating oral diet, ambulating, and pain is controlled with oral medications. The operating surgeon will continue to care for the patient post-operatively but cannot be the surgeon ordering the discharge. If drains are present, they will remain at the discretion of the operating surgeon. If the patient is discharged with drains, these will be removed later in the outpatient setting at the direction of the operating surgeon.

### Outcomes

Primary outcome: Hospital Length of stay (hours).

Secondary Outcomes:

- Patient Reported Outcomes at baseline, 30 (± 14 days), 1-year (± 90) days and 2-year follow-up
  o HerQLes summary score
  o Short-form Patient-Reported Outcomes Measure Information System (PROMIS) Pain Intensity Scale[11] score
  o EuraHS
  o NRS 11 (also scored the 5 following days after surgery)
- Opioid consumption
- Hernia recurrence
- All complications during hospitalization, 30 days, 1-year follow-up postoperatively.
- Wound morbidity
  o SSI
  o SSO
  o SSOPI
- Readmission
- Perform a formal cost effectiveness analysis utilizing quality adjusted life years and incremental cost effectiveness ratios.

### Participant timeline

Estimated patient accrual time is 3–4 years. Data collection will occur over 3 years from randomization of each patient and includes baseline, hospital stay, 30 days, 1-year days follow ups. Details are shown in the Table 1.

**Table 1:** Schedule of enrollment, interventions and assessments

	Baseline	Hospital-stay	30-day	1-Year
Documentation of Inclusion/Exclusion	X			
Informed Consent	X			
Randomization	X			
NRS-1	X	X	X	X
PROMIS 3a SF	X		X	X
HerQLes	X		X	X
Opioid Consumption	X	X	X	X
EuraHS	X		X	X
Direct costs		X		
Composite recurrence				X
Hernia recurrence inventory				X

Scheduled Interventions applied to consented patients during baseline, hospital stay, 30 day and one year follow-up

### Sample size

Using an alpha of 0.05, a power of 0.90, and determining a 24-h difference in length of stay, the necessary sample size is 86 patients in each arm, yielding a total of 172 patients. Estimating 10% attrition at 30 days, 200 patients would be needed, 100 patients enrolled in each arm.

### Recruitment

This is a multi-center trial from selected high volume hernia centers with fellowship trained hernia experts. Recruitment will take place at Cleveland Clinic, University of Tennessee, Prisma Health, and University of Florida. We expect to complete enrollment within 3–4 years.

## Assignment of interventions: allocation

### Sequence generation

Patients will be randomized in a 1:1 ratio using a computer-generated permuted block method with random block sizes. Computer-generated treatment random allocation in REDCap® will randomize robotic or open retromuscular hernia repair in the clinic office after consenting. However, since the scheduling requires prior knowledge of how the surgery will be performed (open or robotic), the operating surgeon will know the randomization arm.

### Concealment mechanism

The randomization table was generated by a statistician and concealed from the study team within REDCap®

### Implementation

Patients will be enrolled during preoperative consultation in the outpatient setting. The allocation sequence will be generated by the research coordinator in REDCap®.

## Assignment of interventions: Blinding

### Who will be blinded

Study participants and the assessor for discharge will be blinded. Additionally, operative notes are blinded to the assessing discharge surgeon. Peri-operative nursing and anesthesia have been educated and precautions taken to decrease the possibility of the patient being unblinded unintentionally. These include direct communication to the anesthesia staff and perioperative nursing the day of surgery. Pre- and post-operatively, a sign is placed in the patient chart and over the bed to inform all caregivers that the patient is in a trial where they cannot be informed of the operative approach.

### Procedure for unblinding if needed

Participants will be unblinded after 6 months, if they inquire about the randomization arm.

### Data collection and management

#### Plans for assessment and collection of outcomes

HerQLes, a validated 12 question survey with an MCID of 15.6, will be collected at baseline and each time point to assess abdominal wall-specific quality of life [12, 13]. PROMIS 3a Pain Intensity Surveys will be collected at all timepoints to assess pain [11]. EQ-5D-5L with visual assessment score will be collected at all timepoints to assess overall quality of life [14]. Hernia recurrence will be measured using a pragmatic definition [15]. Cross sectional imaging will be assessed by three surgeons blinded to the operating surgeon and randomization with recurrence defined by consensus of at least two. In the absence of imaging, a clinical exam will be used to assess recurrence. In the absence of either imaging or clinical exam, the patient response to the presence of a bulge on the Hernia Recurrence Inventory [16]. Patients will also be asked to guess their intervention allocation at 1 year postoperatively.

#### Plans to promote participant retention and complete follow-up

Patients are scheduled for standard of care clinic appointments at 30 days and 1 year and 5 years postoperatively. Patients will be contacted by the research team for patient reported outcomes in the 2-year follow-up window and scheduled for a CT and clinical visit if they report a bulge on the HRI, which is considered standard of care. We will promote complete follow up through multiple approaches. The 30-day follow-up period will extend from 16 to 44 days postoperatively, the 1-year follow-up period will extend from 9 to 15 months postoperatively, the 2-year follow-up period will extend from 18 to 30 months postoperatively, and the 5-year follow-up period will extend from 54 to 66 months postoperatively. Appointment reminders will be sent via patient online portals and nursing phone calls. Virtual follow-up visits can be accommodated, and patients can complete imaging at their local institutions. For patients who have missed follow-up appointments, the dedicated study coordinator, research fellow, and/or surgeon will call patients to reschedule and/or collect patient reported outcomes over the phone. This will increase convenience for patients and protocol adherence. Appropriate statistical analyses will be conducted to account for missing data.

#### Data management

Patient characteristics and operative details will be captured in the ACHQC database, accessed using an assigned username and password. Treatment arm allocation, postoperative complications, CT results, and patient-reported quality of life scores will be collected in REDCap®, a secure electronic database accessed by the investigator and designated study team members using an assigned login and password. Only the principal investigator, research coordinators, and biostatisticians will have access to patient data for routine data quality assessments and data analyses. All electronic records pertaining to the clinical study will be password-protected, and only approved study members listed in the Institutional Review Board (IRB) protocol will have password access.

### Statistical methods

#### Statistical methods for primary and secondary outcomes

All analyses will be conducted on intention to treat basis (ITT). An ACHQC statistician will perform analyses from all participating centers and will require access to identifiable data. A DUA will be executed prior to sharing of PHI. Patient characteristics and operative comparisons will be summarized by the treatment group. Any departures from ITT will be documented and reported.

Participants’ clinical and demographic characteristics will be summarized by treatment arms. Differences between two groups will be assessed using Wilcoxon rank sum tests for continuous variables and Chi-square or Fisher’s exact tests for dichotomous variables. One-sided p-values less than or equal to 0.05 will be considered statistically significant.

### Primary analysis

The primary outcome for this study is length of stay measured in hours. Length of stay will start at the time of surgery start and will end when discharge order is placed. Analysis will be performed using the intent to treat analysis. The comparison in length of stay between two groups will be conducted by using two-sided Wilcoxon ranked sum test with significance level of 0.05. Two sample student’s t-test with log-transfomation would also be considered if we are interested to interpret the length of stay with mean and standard error. Per-protocol analysis will also be done if there’s excessive non-adherence or switching groups during the study. Tipping point analysis will be performed when loss of follow up is anticipated.

### Secondary analyses

NRS 11, PROMIS, HerQles and EuraHS scores will be collected pre-operatively as baseline scores. The difference in mean scores at 30 days and 1 year between two groups will be compared using linear mixed effect models that include the baseline measurement, group by time interaction as fixed effect, and repeated measurements and possibly the surgeon as random effects. The main effect of group will be omitted from the model to force a common baseline effect across groups. The planned linear mixed effect models allow patients to remain in the analysis even when they are missing follow up data. If the distributional assumptions are not met, then alternate generalized linear mixed effect models will be considered.

OR time and Cost would be compared by using the two-sample Wilcoxon ranked sum tests and the cost data would possibly be re-scaled.

SSI, SSO and SSOPI, along with other complications, re-admission and recurrence will be compared by using two sample Chi-square or Fisher’s exact tests.

### Methods in analysis to handle protocol non-adherence and any statistical methods to handle missing data

The primary outcome for this study is length of stay. Analysis will be performed using the intent to treat analysis. The comparison in length of stay between two groups will be conducted by using two-sided Wilcoxon ranked sum test with significance level of 0.05. Two sample student’s t-test with log-transfomation would also be considered if we are interested to interpret the length of stay with mean and standard error. Per-protocol analysis will also be done if there’s excessive non-adherence or switching groups during the study. Tipping point analysis will be performed when loss of follow up is anticipated.

### Composition of the data monitoring committee, its role and reporting structure

There will be a data safety monitoring board (DSMB) for this study. The members of the DSMB will consist of two surgeons and one statistician who are experienced with clinical research. Audits by the DSMB will be performed at their discretion to ensure no significant safety issues arise. All data will be stored in secure databases.

### Interim analyses

No interim looks were planned. No planned subgroup study was identified during the protocol development.

### Adverse event reporting and harms

All adverse events are captured by the research coordinator and reported annually to the IRB per institutional requirements and to the DSMB each time it meets. All serious adverse events (e.g., return to the operating room within 30 days or mesh-related complication, ICU admissions, unplanned readmissions, reoperations) are reported to the principal investigator and DSMB within 24 h of the event occurring.

### Frequency and plans for auditing trial conduct

Research regulatory officers from the Digestive Disease Institute at the Cleveland Clinic Foundation will audit trials to ensure regulatory compliance. Other sites that are brought on will have a planned audit after their first five patients enrolled, or after their first 6 months of enrolling, whichever comes first.

### Ethics approval and consent to participate

Cleveland Clinic Foundation Institutional Review Board #22–945. Written, informed consent to participate will be obtained from all study participants.

### Plans for communicating important protocol amendments to relevant parties (e.g. trial participants, ethical committees)

Protocol modifications will be submitted to the study on the Cleveland Clinic IRB for approval and communicated to other sites within 10 days for modification to their institutional IRBs. Re-education regarding significant protocol amendments will be directly communicated to each sites PI.

### Who will take informed consent?

Informed consent from potential trial participants will be obtained by study investigators or research personnel during the preoperative visit depending on the sites standard research practices.

## Discussion

Robotic retromuscular hernia repairs have been shown to be safe, effective, and potentially beneficial to patients compared to open techniques [3, 5, 6, 8, 17, 18], but still lacking high quality data. Recently, a randomized trial comparing robotic to open abdominal wall reconstruction for ventral hernias was published [4]. There are some important limitations to this trial including the primary outcome being composite wound morbidity in early post-operative period [1]. These cohort of patients have a planned long term follow up which will be interesting to see if any benefit exists to having a minimally invasive approach to abdominal wall reconstruction. This addition of a randomized trial adds significant value to the current body of literature in the field.

Other retrospective data currently exists. A large case control study found no difference in intraoperative complications between robotic and open TAR (8.9% vs 16.5%, *P* = 0.137) and an 8.9% conversion rate to open. Yet other retrospective series highlights lower estimated blood loss, but without difference in transfusion requirements [6]. Carbonell and colleagues published a 2:1 propensity score matched cohort study of robotic and open retromuscular ventral hernia repairs from the ACHQC in 2018 [3]. This data demonstrated a significantly higher SSO incidence in the robotic group, which was driven by a higher rate of seromas not requiring intervention. There was noted to be a decreased hospital length of stay. This benefit seems to be a recurring consistent advantage of the robotic approach when compared to open retromuscular ventral hernia repairs in retrospective studies [1, 3, 5, 6]. The remaining studies published have been case series, systematic reviews, or large registry data, all of which have significant limitations and inherent bias due to trial design [2].

Based on the available literature, we designed a randomized, double-blinded trial comparing robotic to open ventral hernia repairs using retromuscular techniques. We hypothesized that robotic retromuscular repairs will be superior to open by reducing length of stay by at least 24 h compared to open repairs. This will provide high-quality data to help surgeons make important clinical decisions regarding the care they provide for patients.

## Trial status

Protocol version: September 2024 Version 5.

Recruitment began July 25, 2022, and is anticipated to be completed in July 2025.

## Data Availability

The trial status can be accessed on ClinicalTrials.gov using the study identifier number. The data are publicly available for review in accordance with the platform's policies and guidelines.

## Abbreviations

SSO: Surgical site occurrences
SSI: Surgical site infections
SSOPI: Surgical site occurrence requiring procedural intervention
HRI: Hernia recurrence inventory
HerQLes: Hernia-Related Quality-of-Life Survey
IRB: Institutional Review Board
DSMB: Data Safety and Monitoring Board
TAR: Transversus abdominis release
RCT: Randomized controlled trial
CT: Computed tomography
ACHQC: Abdominal Core Health Quality Collaborative
REDCap®: Research Electronic Data Capture
